# From general rejection to individual normalization: Ambivalences in discourses on intimate partner violence by young Spaniards

**DOI:** 10.1371/journal.pone.0310745

**Published:** 2024-09-24

**Authors:** Ismael Ocampo Bernasconi, Eva Espinar-Ruiz, Daniel La Parra-Casado, Carmen Vives-Cases

**Affiliations:** 1 Doctorate Program of the Gender Studies Institute, Alicante University, Alicante, Spain; 2 Department of Sociology II, University of Alicante, Alicante, Spain; 3 Department of Community Nursing, Preventive Medicine and Public Health and History of Science, University of Alicante, Alicante, Spain; 4 CIBER of Epidemiology and Public Health, Madrid, Spain; University of Salerno, ITALY

## Abstract

Violence against women continues to be a serious social and public health problem all over the world, despite its high level of social condemnation. The aim of this study is to include the concept of ambivalence in order to analyze the perceptions that young people have of intimate partner violence (IPV). We conducted a qualitative study based on 20 semi-structured interviews with young Spaniards (men and women) who were segmented according to involvement or not in activism against IPV. A critical discourse analysis was performed based on situational, attitudinal and temporal ambivalences. The results show the presence of ambivalent discourses that express a high level of condemnation towards IPV and, at the same time, justify certain violent practices when considering the situational framework. This leads to discursive contradictions when considering jealousy in a couple’s relationship, the responsibilities of violence that women suffer and the social origin of IPV. These ambivalences are influenced by the social context, as those involved in activism against IPV express a more coherent discourse that does not justify or accept any kind of violence. Nonetheless, non-activists represent ambivalences in their attitudes towards IPV studied in this research. The results suggest the need to reflect on the strategies used with young people to eradicate IPV by considering the ambivalent nature of attitudes.

## Introduction

Intimate partner violence (IPV) is a social and public health problem that occurs in all cultures, countries and social classes [1]. Furthermore, 58% of murdered women all over the world are killed by a family member and 34% by their partners [2]. More than one in every four women over the age of 15 reports to have been a victim of physical or sexual violence at some point in their lives by an intimate partner [3].

This kind of violence also prevails in younger populations. Thus, approximately 16% of young women all over the world between 15 and 24 years of age state to have been victims of physical or sexual violence in their intimate relationships in the past 12 months. This represents the highest percentage for this period of time among the different age groups [3]. In addition, several studies have identified the prevalence of other forms of violence towards young women, such as controlling behaviors or psychological violence [4, 5]. There is evidence that young people seem to be more tolerant than adults towards controlling behaviors in relationships, as they accept and normalize them [6]. Beliefs related to romantic love myths come into play, as people relate jealousy to love [7, 8], which leads to it being accepted and interpreted as inherent to intimate relationships. Normalizing controlling behaviors in relationships is associated with a higher tolerance towards IPV by men who, in different contexts, continue to accept and normalize street harassment and to objectify women’s bodies in public spaces. In this sense, men linked to activism against IPV are expected to have more egalitarian positions. For instance, they are more likely to intervene against violent or offensive behaviors of other men towards women [9]. They also avoid not reproducing sexist and misogynistic beliefs [10, 11], and also, they defend equality in gender relations [12].

The evidence reflected by the data on the prevalence of different kinds of IPV seems to contradict the survey data about attitudes towards it. For instance, 84% of EU citizens consider violence against women to be unacceptable [13]. Furthermore, 78% consider there to be a high frequency of violence against women [14]. In the specific case of the young Spanish population, they are able to recognize and be aware of the different forms of direct violence against women but, for example, not other related aspects, such as objectifying their bodies. Ambivalent cultural frameworks are promoted for women, where their sexual freedom and bodily empowerment are fostered. However, there are a number of traditional imaginations whereby their bodies are controlled [6, 15]. In a manner, tension is experienced by young people when building their affective-sexual relationships, while having to live with contradictory imaginaries that combine traditional and modern models on how men and [16–18] women should be [6, 19, 20]. This supports the fact that young people express contradictory discourses on IPV.

In light of this context, the study in this article explores how a group of Spanish young people perceive different forms of IPV. Our objective is to contribute to the knowledge about the attitudes that the Spanish youth have towards different forms of IPV in affective-sexual relationships using the category of ambivalence. This concept has been approached in a variety of ways throughout history, therefore first we must define and shape it for this research. In order to do so, different theoretical perspectives are considered that directly or indirectly address the concept of ambivalence. In this sense, the aim of this study is to include the concept of ambivalence to analyze the contradictory perceptions that young Spanish people have regarding IPV.

## Theoretical axis—Ambivalences

### Conceptualization

The concept of ambivalence has been addressed from psychoanalytic approaches [21], structural functionalist [22], behavioral psychology [23] and postmodern studies [24]. Thus, ambivalence is shown as a concept with different disciplinary perspectives, yet it is always related to what is considered to be ambiguous, uncertain and contradictory.

In the last decades, there have been attempts to define the concept by highlighting its broad level of complexity. For instance, Romero Morales [25] explains that ambivalence constitutes the ability to discursively come and go based on an idea or its opposition. Therefore, it is an action that is not necessarily contradictory, but rather it is experienced as multiple and dynamic. Colli and Neto [26] understand ambivalence based on the tension that human beings experience between what was and what will be; a tension framed by living with the past, present and future. In this context, ambivalence is related to a space-time-multiple concept in which people are immersed.

From a sociological perspective, ambivalence has been approached in an indirect way. The seminal work by Georg Simmel [27] in 1908, while focusing on the situational status, demonstrates the existence of dualism since the subject is a social and individual being at the same time. Simmel’s approach does not speak of a double existence, but an ambivalent one, insofar as both aspects of human experience coexist. In this way, the subject must face non-coherent situations with different meanings, which forces them to take on contradictory attitudes, as was already describe in classic contribution as Goffman’s work [28]. This self must have a certain level of consistency to give the impression of continuity, seeking to deny contradictions. Society forces individuals to be self-consistent while promoting a complex and varying social reality that constantly leads to the inconsistency of that self.

The self is constituted by the conjunction of multiple signifiers and social imaginaries that represent a subject formed by multiplicity and fragmentation [27]. However, this multiplicity is based on the social context that determines it, to the extent that some contexts promote more plurality than others. Societies that present a higher level of differentiation and complexity also express it internally in each subject [29]. Thus, the self is always constituted in relation to the situation, generating an internally complex structure where different logics overlap [30].

In this study we define ambivalence as multiple representations, beliefs and attitudes towards social facts. Ambivalence can be translated into ambivalent actions, attitudes, beliefs and/or feelings. In other words, although the origin of ambivalence is external to the subject, it emerges and is displayed in people’s experiences. Ambivalence is always something that human beings encounter when interacting between what is individual and social.

### Ambivalence in IPV studies

Ambivalence is directly and indirectly present in IPV studies. In terms of it being directly present, the conceptualization of ambivalent sexism has been essential [31]. It raises the fact that beliefs of sexism are marked by ambivalent ideas of non-uniform antipathies, which are intermingled with benevolent notions about a discriminated group, in this case women. Ambivalent sexism is constituted by direct hostile attitudes and apparently positive attitudes that ultimately limit the possibilities of women, putting into play a male over women domination strategy.

Likewise, ambivalence has been indirectly present in various research and analyses of IPV and gender relations, although without being named as such. Thus, the way traditional and modern values, attitudes and beliefs on gender roles coexist have been identified. For instance, this has been theorized by Marcela Lagarde [32], who introduces the concept of *gender syncretism* to refer to the pressure that contemporary women experience by having to fulfil the traditional housewife role alongside the modern elements of an emancipated woman. This logic of analysis can be observed in studies on the figure of the modern mother [33, 34] or when analyzing how women’s sexuality enters a contradictory framework, insofar as traditional beliefs collide with new imaginaries about the empowerment of the female body and sexual freedom [15]. The consequence is an impossible balance for young women to achieve as they are socially punished for everything they do [6].

In the case of masculinity studies, Bridges and Pascoe [35] perform a bibliographic review on the concept of hybrid masculinities, reflecting how several studies identify the coexistence of different gender values and beliefs in the imaginaries of masculinities. Within this line of research, other studies [36, 37] identify hybridity between the traditional and modern models of masculinity. However, they are far from leading men towards equality, as they reinforce the male domination system by enabling domination by men over women [38]. Finally, other researchers have identified ambivalences among young people when assessing IPV globally and situationally. These studies identify discourses where a general knowledge of IPV and its causes can be observed; and a clear condemnation can be seen. However, when the analysis is based on individual situations, certain forms of violence in couple relationships are justified. For instance, the possibility that women can be violent in self-defense [39]; that male violence is validated when being protective [7]; or normalizing daily sexist behavior and gender violence [40].

These studies highlight the contradictions in beliefs about masculinity, femininity and IPV, making clear to us the need to incorporate ambivalence as a category of analysis to understand current processes of change, marked by contradictions, tensions and ambiguities.

## Methodology

The information gathered for this article is part of the PositiveMasc project that has the aim of analyzing the beliefs and attitudes of young people towards IPV and the social and cultural constructions of masculinities in Israel, Ireland, Sweden and Spain. The aim is to develop strategies to include men as important agents in fighting against IPV [41]. In order to do so, qualitative research was conducted including interviews and focus groups with young people and stakeholders. Only information collected on Spanish experiences is used in this article. In order to achieve the objective of analyzing the discursive ambivalences of young people, only individual interviews with the young people conducted in 2019 are analyzed.

### Sample characteristics

The interviewees included 20 young people (11 men and 9 women) aged between 18 and 24 years, from Alicante and Madrid. The participants were all cisgender men and women with different sexual orientations (2 men and 1 woman identified as LGTBI). Furthermore, different levels of education were included (2 men and 3 women without higher education [HE]). Young people especially involved in different forms of activism against IPV [AAIPV] were sought (2 women and 3 men activists) in order to identify particularities of their discourses on the topic and compare them to non-activists. Table 1 presents the demographic details of the sample.

**Table 1:** Sociodemographic information of the participants.

Interview	University	Sexual orientation	Involvement in activism	Gender
1	No	Heterosexual	No	Woman
2				
3				
4	Yes			
5				
6		Bisexual		
7				
8		Bisexual	Yes	
9				
10	No	Heterosexual	No	Man
11				
12				
13	Yes			
14		Bisexual		
15		Homosexual		
16		Heterosexual	Yes	
17				
18	No	Homosexual		
19	Yes			
20		Bisexual		

Table modified from the original previously published in another article of the PositivMasc project [12]

The participants were recruited by handing out leaflets in education centers, NGOs and institutions that work with young people or sharing on social networks. They were informed on the aims of the research and that their identities would be concealed when using the information.

### Procedure

The interviews were semi-structured where the interviewers used an initial script to discuss the different topics. They were conducted in Spanish and lasted approximately an hour and an hour and a half. The structure of the script was divided into three large blocks. The first block addresses the perception of gender, femininity, masculinity and IPV in a general way, with questions that enabled an open dialogue on each of the topics. The second block focuses on working on a series of vignettes on IPV situations (Table 2). Finally, the third block contains closed-ended questions in order to identify strategies to involve young men and women as transformation agents to eradicate IPV.

**Table 2:** Description of the vignettes used in the interviews with young people regarding IPV in Spain (2019).

Vignette	Description
Digital violence	In a heterosexual couple, the girl begins to receive lots of likes on social networks from another boy. Her partner asks about it and who the boy is, he asks her to block him and to give him her social network passwords.
Violence on social networks/Revenge	In a heterosexual couple, the girl ends the relationship and the boy insists on wanting to get back together, despite the fact she has repeated several times she does not want to. Then, he threatens that he will share intimate photos that she shared with him when they were together.
Emotional violence	A heterosexual couple have been together for 3 years. They have recently started to argue a lot and he has insulted her repeatedly. So, she does not want to have sexual relations with him and he suspects she is cheating on him.
Physical violence	Continuing with the previous example, and in this case, he has slapped her when arguing as he believes she is not being faithful.
Sexual violence	Continuing with the previous example, now he insists on having sexual relations and he says that if she really loves him she should have sex with him.
Sexual harassment	At a party, there is an outgoing girl who likes to wear short skirts and tight tops, a boy passed her and grabbed her bottom without her consent.
Rape	That same girl after the party is walking alone, a bit drunk. She is harassed by a group of men who rape her.
Alternative masculinity	A group of friends are at a nightclub, one of them is insistently harassing a girl who has repeatedly asked him to leave her alone and to not touch her. His friends encourage him to keep harassing her, except for one, who intervenes and tells him to leave the girl alone. The latter is rejected by his entire group of friends.

Participation was voluntary and participants had the option to withdraw from the study at any time. Written informed consent from the study participants was obtained prior to enrolment. Confidentiality was guaranteed in adherence to the European Union General Data Protection Regulation (2016/679). The study was approved by the ethical committee of the University of Alicante, Spain (reference number UA-2019-04-15).

### Information analysis

The interviews were analyzed based on the proposal of critical discourse analysis (CDA), seeking to link micro perspectives with macro perspectives, addressing what is singular as a whole and in relation to what is considered to be social [42]. In this sense, CDA breaks the notion of rigidity and uniqueness and incorporates the interaction of the multiple contexts, norms, rules and power in the analysis of the discourses [43].

The analysis process to address ambivalences included the following stages: First of all, we used Atlas.Ti program to code the interviews. Based on the bibliographic review of IPV in the young population in Spain, four discursive axes were identified: (*i*) the origin of IPV, (*ii*) condemning violence, (*iii*) women’s body and sexuality and (*iv*) control and jealousy. Afterwards, we analyzed each interview by examining all the codes related to the four discursive axes in order to identify ambivalences and coherence. Subsequently, the results of the ambivalences and coherence of each discursive axis were combined in all interviews. Finally, the results for each discursive axis were analyzed according to three types of ambivalence. These three types arise jointly from the analysis and the theoretical review.

The first type of ambivalence is situational ambivalence. This type of ambivalence is constructed based on the proposals by Simmel [27] and Goffman [28]. They suggest that subjects may find themselves faced with the incoherence of having to relate different meanings to the same facts, contradicting social status with individual status. Thus, this ambivalence is focused on the presence of ambiguous or contradictory meanings that occur between the perception of a specific situation of IPV (situational perception) and the general concept of IPV.

The second category is Attitudinal ambivalence, which is constructed based on the work of Glick and Fiske [31] and Thompson et al. [23]. It is understood as the presence of a double valuation (negative and positive) towards something. People can have incoherent feelings towards specific topics. This arises from the multiple beliefs which are expressed both in emotional forms and based on rationalization. Temporal ambivalence is the third category and refers to the valuation of a fact or situation influenced by the coexistence of two or more historical contexts, thus generating the presence of different values and beliefs towards the same fact [26]. The presence of different time frames coexisting in the construction of social imaginaries leads to the possibility of generating tensions on how social relations are understood (in this case, gender).

A fourth category of analysis is added by also paying attention to coherent discourses that emerge when there is a lack of ambivalence. In other words, it is present when participants have a uniform discourse towards a fact or situation. The analysis of this coherent discourse has two purposes. On the one hand, it helps to identify which aspects of IPV are most clearly perceived by young people. On the other hand, it helps to understand and analyze the ambivalent discourse through comparison.

Throughout the analysis, particular emphasis was placed on the differences between the discourses of activist and non-activist participants, as it was identified that inhabiting spaces of activism against IPV intervenes in the presence of ambivalence.

The analysis process was firstly conducted by the first author, who coded and categorized the information. The other three researchers took part in the second part of the analysis by reinforcing it and writing the article. The final results emerged from dialogue and feedback by all four authors.

## Results

The analysis of the interviews was based on the typology of ambivalences. In each one, the four discursive axes on IPV were incorporated as shown in Table 3. In order to highlight the discursive ambivalence raised at different points in the interviews an underscore in parentheses was used.

**Table 3:** Ambivalent discourses on intimate partner violence (IPV) in young Spaniards (2023).

Discursive axes on IPV	Types of ambivalences			Coherent discourses
**Origin/responsibility of violence**	**Situational:** young non-activists (men and women) and activist boys understand the social origin of IPV, but they tend to individualize the responsibilities of violence.			Sexist culture is understood as the origin of violence by activist girls. Violence is personalized by non-activist men.
**Accepting and condemning violence**	**Attitudinal:** young non-activists minimize psychological violence in comparison to physical violence, despite condemning all kinds of violence.	Regarding verbal violence, certain kinds of insults are accepted.	**Temporal:** young non-activists accept violence in private settings, but not in public.	They condemn the misuse of intimate photos of the couple. Activists condemn all kinds of violence.
**Control and jealousy in intimate relationships**	**Situational:** young non-activists socially condemn jealousy, but they accept or understand jealousy in individual cases or in their own life experiences.	**Attitudinal:** young non-activists condemn jealousy, but they accept it when faced with physical violence. Violence is accepted in private settings, but not in public.	**Temporal:** ideas of complementarity in couples that justify jealousy, alongside ideas of no control in relationships in young non-activists.	Women and men activists do not justify jealousy or control in couples in any case.
**Women’s body and sexuality**	**Situational:** young non-activists with a general discourse on women’s rights, but blame them for the violence they receive because of their attitudes Non-activist women accept	non-consent in their relationships, but condemn it in general. **Attitudinal:** young non-activists consider violence differently depending on the level of formality that the couple has.	**Temporal:** presence of sexist beliefs on women’s body and sexuality, while coexisting with modern imaginaries of a free and empowered woman in non-activists.	Activists do not blame women in any case for the violence they receive.

### Situational ambivalence

When analyzing situational ambivalence, it was possible to observe discourses where contradictions or ambiguities were generated when contrasting the way of thinking regarding general and situational forms of IPV. An example of this situation emerged in young non-activists who presented ambivalent discourses about jealousy and controlling friendships. Considering that a condemnation of jealousy was expressed, it was understood as a form of violence, while also being perceived as unavoidable in intimate relationships at the same time.

*Absolutely nobody should have the authority to decide for another person, whether a man is with a woman, a man is with a man or a woman with a woman, it does not matter, it is simply the rights people have. (_)It is something that someone cannot avoid and, if you, if you reach a point where it is really hurting you to see that person liking a post, I understand that you need to have a conversation to ask your girl who that boy is* (Non-activist Man).

Jealousy was perceived as a way of controlling to isolate the other person and reducing their ability to be autonomous. However, at the same time, jealousy was accepted as it was understood as unavoidable and normal in relationships. A fear of infidelity was an important justification of it. Social networks have a key role in this sense, as control in relationships occurs by not trusting interactions within said space. To this point, in many of the interviews, comments based on personal experience were expressed, generating situational ambivalences by contradicting what they understand as correct in the social sphere.

Likewise, something similar happens to what was reported in the last example when talking about sexual consent. We could see in non-activist women that, despite strongly positioning themselves on the need for consent in sexual relations in an intimate relationship, they naturalized the fact of giving in to pressure to have sexual relations as a way of not causing conflict. It was not seen as a violent sexual act as seen in the interview below.

*It can be hinted, but then having it, I do not know, I would say no. A sexual relationship always has to be between two people, if it is not, it is not a consensual relationship. (_) Sometimes you give in, even if you do not want to, you end up doing it because it is like “oh…”. Yes, I have seen it* (Non-activist Woman).

Another example occurred when discussing the causes and responsibilities of violence, where non-activists and activist men expressed ambivalent discourses that demonstrate recognition the social origin of violence, but place the main responsibility on the person who carries it out. The following interviewee gives an example of this.

*Who are the people responsible? Everything comes from a point that goes much further back, from the education you have been given to the values you have had, what you have been able to see within your family or around you, even in the neighborhood where you have lived, everything can influence you to generate gender violence.* (_) *I think that Gustavo feels insecure and that is why he asks María to eliminate any person from her surroundings who could be a competitor or someone who can take away time, love or affection from him* (Non-activist Woman).

### Attitudinal ambivalence

In addition to situational ambivalence, other types of ambivalence were observed in the discourses, as is the case of attitudinal ambivalence where differentiated valuations were made depending on the fact being explored. A generalized example by young non-activists was to minimize verbal violence when compared to physical violence. In this case, they generally understood that IPV was never acceptable in all its senses. However, when assessing the different kinds of violence, they tended to establish a hierarchy, classing physical violence as intolerable. Verbal violence, which was perceived as less serious than physical violence. This discourse was repeated in non-activist women and men.

*No, I do not think it is justified. Honestly, I do not want this to sound bad, but I could understand insulting another person. I understand that it is something that hurts, realizing they are cheating, for example, it hurts and when you are angry you can say “hey, you are this or you are that” and you insult them, but then hitting them, no, no, I do not think that is justified* (Non-activist Woman).

Therefore, certain types of insults were understood to be tolerable as part of verbal violence, as they were considered to be a daily part of relationships. This type of differentiated assessment was also marked by the scope in which IPV is expressed. Non-activist men and women understood that IPV is intolerable in public spaces or in the presence of other people, but they could accept or understand that verbal violence occurs in private or intimate settings as expressed in the following excerpt.

*There is no reason to hit another person (_) Uff. No, and if it is done, do it in private, not in public. Not even insulting, I am not sure, saying “you have been cheating on me, you are an idiot”, whatever, do it in private, not in public”* (Non-activist Man).

The interviewees made differentiated assessments according to the different characteristics that violence could have regarding the modality and scope. These ambivalences, which reflected contradictory attitudes, were mainly influenced by temporal ambivalence, as seen in the following section.

### Temporal ambivalence

In the case of this kind of ambivalence, young non-activists were identified to simultaneously have modern and traditional values on different analysis axes. An example of this was the discourses on women’s sexuality and freedom in relationships. In the case of female sexuality, young non-activists were ambivalent when expressing a discourse on women’s rights, while blaming them for the violence they receive because of their attitudes, ways of dressing or drinking alcohol.

*I do not think she is to blame but, in this case, it is a girl, but it does not matter if it is a boy or a girl. So, everyone is free to dress however they want, however they feel comfortable and that does not mean someone else has the right to touch you (_). But what is true is that you have to be aware, nowadays, the way things are and so… I do not justify it, but, hey, if you dress like that nowadays, the way things are, it is true that you have more chances of something terrible happening* (Non activist Woman).

Here the interviewee expressed that a woman should not be attacked because of the way she dresses or how she is behaving, but at the same time the person questioned the context or the way the victim dresses. This ambivalence was also present in the beliefs that non-activists have about intimate relationships when they expressed discourses about freedom in them, regarding the right that each person has to have the friends they want without the counterpart intruding. Yet, at the same time, they accepted control over friendships based on the idea of complementarity in intimate relationships, as expressed by the following interviewee.

[Decisions in the relationship should be made] *mutually or one person sacrificing something, knowing that there will be retribution. I do not know if I am explaining myself. Everything has to be decided by the parties, or one of them, like any type of relationship, there are two in a relationship, one has to be the one that gives more and another the one that pushes more (_). A relationship is not being a slave to the other. I understand the fear, but you also have to understand, like you want to go out with your friends, you need to let your partner go out with their friends* (Non-activist Man).

The notion of complementarity ends up becoming a justification for control which shows the presence of two different temporal models in discourses on relationships: a modern one in which each of the parties is open to independence, and a traditional one in which each party must give up and lose aspects of their individuality.

### Coherence

In contrast to the aforementioned ambivalences, some coherent discourses regarding IPV were also been observed. The majority of them were by activists who completely condemned violence, understanding the social character that sustains it and being significantly aware of the topic. In particular, women activists did not hold women responsible for the violence they receive, did not justify jealousy or control in intimate relationships and they did not individualize the causes and responsibilities of IPV. Another coherent discourse, but in this case contrary to that of the women activists, was by non-activist men, both university students and not. The interviewees argued that the only person responsible for violence is the one who is violent, without mentioning or identifying social or cultural factors, as observed in the following excerpt.

*I do not know if we can say it is an illness, but I would say so. Jealousy is an illness, but what is… Yes, I would say it is an illness* (Non-activist Man).

Likewise, it is common for non-activist men interviewees to equate violence by men to that by women.

*The person who attacks, rapes, more than a person, whether man or woman, is a rapist. I do not identify the person as a man or a woman* (Non-activist Man).

Thus, these men showed the only discourse without the presence of ambivalence among the young non-activists. The construction of this discursive ambivalence in the young people interviewed will be discussed in depth below.

## Discussion

The analysis developed on the three kinds of ambivalences in the discourses on IPV among young Spaniards enables us to understand the complexity of their discourses regarding this topic.

In this sense, situational ambivalence allows to identify, as observed in other research [7, 39, 40], that young people have contradictions about IPV in general terms, but they also normalize and justify it when discussing specific cases. Therefore, situational ambivalence is displayed regarding jealousy and sexual consent.

In the case of jealousy, situational ambivalence appears in discourses where, on the one hand, the right to freedom and individuality is valued but, on the other hand, control is justified and accepted. This occurs in a general discourse on jealousy to individual cases as seen in the examples in the vignettes, as well as the interviewees’ life experiences. The prevalence of jealousy in affective-sexual relationships among the young population has been identified in various studies, considering that young people continue to believe jealousy is a proof of love [8, 9]. Without necessarily being identified as a form of violence, jealousy is normalized and naturalized as part of intimate relationships [44].

Jealousy is a great example of the close relation between the three types of ambivalences analyzed in this article. Thus, situational ambivalence is observed as there are contradictions between the value placed on what is social and what is individual; attitudinal ambivalence is considered when jealousy is related to verbal and psychological violence that are minimized when in comparison to physical violence; and, finally, temporal ambivalence is related to traditional beliefs about control in intimate relationships coexisting based on the notion of complementarity, alongside modern discourses on individual autonomy. The interconnection between the different kinds of ambivalences can help to understand the complexity in discourses among young people regarding jealousy in their relationships [45].

As previously mentioned, temporal ambivalence has been studied the most in research on IPV. In this article, temporal ambivalence takes up proposal of gender syncretism [32], on the double social valuation regarding women, as two opposing imaginaries coexist about how a woman should be. This fact was also portrayed in the discourses by the young non-activists who clearly stated the fact that women have the right to dress as they wish, drink alcohol and have fun without being questioned. However, at the same time, they are blamed for the violence they receive due to the same aforementioned rights. The ambivalence of two social imaginaries coexisting about what is feminine carries the danger of continuing to justify the violence that women receive. In this sense, we also identified attitudinal combined with temporal ambivalence in these discourses. This fact can be understood as a new form of ambivalent sexism [6, 31]: women’s capabilities are valued, in terms of their bodily autonomy, but at the same time, they are harassed and judged for that same autonomy, maintaining contradictory imaginations towards them.

Another way temporal ambivalence is expressed through the beliefs about intimate relationships where two opposite models coexist about how affective-sexual relationships among young people should and can be. These contradictions in social imaginaries about intimate relationships and how women should behave can promote the presence of a double tension among young people and favor situational ambivalence, as there are incoherences between the general and individual way of thinking. The presence of ambivalent imaginaries on IPV can promote that certain violent practices are not recognized and, therefore, continue to be perpetuated.

On the other hand, when observing the relationship between coherent and ambivalent discourses, we find that it is the social context in which the subject is immersed that generates ambivalences or not. These results show that ambivalent discourse is related to the subject’s experience in a social world with the different layers of significance [27]. For this study, the fact of being linked or not to activism on gender issues is a compelling determinant for the presence of ambivalences above the other variables included in the research (sex/gender and being or not a university student) that did not provide significant results. It could be inferred, like in other research [11, 12] that works with men who are activists in gender equality, that being an activism allows through practice a deeper understanding of the problem of IPV.

In the same sense, coherent discourses were also greatly present in non-activist men and the personalization of the responsibilities of violence. They understand that IPV is a reprehensible act, but they fail to see it as a social problem, as already observed in other studies [11, 45–47]. These results suggest two directions need to be developed to work on with men. On the one hand, emphasis must be made on the social and historical nature of IPV in order to break the personalist perspective. This entails the risk of understanding violence as a problem that is solely the responsibility of the person exercising it. On the other hand, strategies need to be designed that include them as transformation agents, making them an active participant to eradicate IPV, bringing them to activist spaces and, in this sense, incorporating a situated experience that sensitizes them on the problem. The important question here is how we can create conditions for young people to become activists? The answer to it should be explored in future research.

## Limitations

This research aims to value the use of the ambivalence category to analyze attitudes towards IPV. However, its scope is limited to the context, Spain, and the characteristics of the sample. It is necessary incorporating other axes for future research of social inequality among its variables, such as: migratory status, social and economic level, ethnic group or people with disabilities.

On the one hand, some of the results should be taken with caution, regarding coherence found in the activist profile. It would be a mistake to think that being an activist on gender topics exempts said people from violence, as what is being analyzed in this article is the attitude towards the topics, not exercising it. Therefore, the results are limited as they only refer to the discourse. In future, studies should also include practices.

## Conclusions

Understanding the characteristics of ambivalence category and its relation to the attitudes in the younger population on IPV has shown to be useful, as it allows us to understand the complexity and contradictions of the issue. The presence of knowledge on a topic does not mean that one has a certain position on it when it is discussed in a particular case. In this sense, conceptualizing situational ambivalence allows for including a category of analysis for the presence of these discourses. Likewise, attitudinal ambivalence helps to understand how differentiated valuations can be given to violence, where certain forms of violence are tolerated and accepted and others condemned. Attitudinal ambivalence is combined with temporal ambivalence, as the social imaginaries of the opposite gender belonging to different historical contexts of today coexist, thus sustaining contradictory attitudes towards IPV. However, the coherent discourse analysis has allowed us to reflect on the weight of the social context in which young people are immersed when contradictory attitudes towards IPV are produced.

Based on these results, it is understood that in order to work with young people it is necessary to consider the contradictory frameworks in which they live in order to design an intervention strategy that takes into account the tensions when ambivalent signifiers coexist on IPV. Therefore, it is expected that the results of this study will enable tools to be designed to implement public policies with the aim of eradicating IPV in young populations.
